# Factors associated to suicide risk in *stricto sensu* postgraduate students: a cross-sectional study*

**DOI:** 10.1590/1518-8345.4590.3460

**Published:** 2021-06-28

**Authors:** Evelyn Kelly das Neves Abreu, Samira Reschetti Marcon, Mariano Martínez Espinosa, Moisés Kogien, Marília Duarte Valim, Frantielen Castor dos Santos Nascimento

**Affiliations:** 1 Universidade Federal de Mato Grosso, Faculdade de Enfermagem, Cuiabá, MT, Brazil.; 3 Universidade Federal de Mato Grosso, Departamento de Estatística, Cuiabá, MT, Brazil.

**Keywords:** Suicide, Risk, Risk Factors, Students, Nursing, Graduate Education, Suicídio, Risco, Fatores de Risco, Estudantes, Enfermagem, Educação de Pós-Graduação, Suicidio, Riesgo, Factores de Riesgo, Estudiantes, Enfermería, Educación de Posgrado

## Abstract

**Objective:**

to analyze factors associated to suicide risk in postgraduate students.

**Method:**

a cross-sectional analytical study, developed with 565 *stricto sensu* postgraduate students from August to September 2019. Data collection took place using a validated instrument containing demographic, socioeconomic, health and academic variables; as well as variables of Module C of the Mini International Neuropsychiatric Interview (MINI), version 5.0; of the CAGE (Cut down, Annoyed by criticism, Guilty and Eye-opener) questionnaire; and the Depression, Anxiety and Stress Scale (DASS-21). Descriptive and multiple statistical analysis was performed using the Poisson regression model, with a significance level of 5%.

**Results:**

40.8% prevalence of current suicide risk. The following variables were associated to current suicide risk: age > 30 years old (p=0.029), absence of faith (p=0.015), depression (p<0.001) and anxiety (p=0.018) symptoms, use of psychotropic drugs during the course (p<0.001), not having a meaningful and inspiring academic work (p=0.013), not having a good relationship with colleagues from the postgraduate school (p=0.033), having family relationship impaired by the demands of the postgraduate school (p=0.036) and concern about the financial situation (p=0.048).

**Conclusion:**

a high prevalence of current suicide risk was identified among postgraduate students, as well as a significant association of this risk with demographic, socioeconomic, academic and health variables.

## Abstract (pt)

**Objetivo:**

analisar os fatores associados ao risco de suicídio em estudantes de pós-graduação.

**Método:**

estudo transversal analítico, desenvolvido com 565 pós-graduandos stricto sensu de agosto a setembro de 2019. A coleta de dados ocorreu por meio de um instrumento validado contendo variáveis demográficas, socioeconômicas, de saúde e acadêmicas; do módulo C do *Mini International Neuropsychiatric Interview* (MINI), versão 5.0; do questionário CAGE (*Cut down, Annoyed by criticism, Guilty e Eye-opener*) e Escala de Depressão, Ansiedade e Estresse (DASS-21). Realizou-se análise estatística descritiva e múltipla com modelo de regressão de Poisson, nível de significância de 5%.

**Resultados:**

prevalência de 40,18% de risco de suicídio atual. Associaram-se com risco de suicídio atual as variáveis idade >30 anos (p=0,029), ausência de prática de fé (p=0,015), sintomas de depressão (p<0,001) ansiedade (p=0,018), uso de psicofármacos durante o curso (p<0,001), não ter um trabalho acadêmico significativo e inspirador (p=0,013), não ter uma boa relação com colegas da pós-graduação (p=0,033), ter relacionamento familiar prejudicado pelas demandas da pós-graduação (p=0,036) e preocupação com a situação financeira (p=0,048).

**Conclusão:**

identificou-se alta prevalência de risco de suicídio atual entre pós-graduandos e associação significativa deste risco com variáveis demográficas, socioeconômicas, acadêmicas e de saúde.

## Abstract (es)

**Objetivo:**

analizar los factores asociados al riesgo de suicidio en estudiantes de posgrado.

**Método:**

estudio analítico transversal, desarrollado con 565 estudiantes de posgrado *stricto sensu* de agosto a septiembre de 2019. La recolección de datos se realizó mediante un instrumento validado que contiene variables demográficas, socioeconómicas, de salud y académicas; Módulo C de la *Mini International Neuropsychiatric Interview* (MINI), versión 5.0; el cuestionario CAGE (*Cut down, Annoyed by criticism, Guilty e Eye-opener*) y la Escala de Depresión, Ansiedad y Estrés (DASS-21). El análisis estadístico descriptivo y múltiple se realizó mediante el modelo de regresión de Poisson, con un nivel de significancia del 5%.

**Resultados:**

prevalencia del 40,18% del riesgo actual de suicidio. Las siguientes variables se asociaron con el riesgo de suicidio actual: edad >30 años (p=0,029), falta de fe (p=0,015), síntomas de depresión (p<0,001) ansiedad (p=0,018), uso de psicofármacos durante el curso (p<0,001), no tener un trabajo académico significativo e inspirador (p=0,013), no tener una buena relación con los compañeros de posgrado (p=0,033), tener una relación familiar deteriorada por las exigencias del posgrado (p=0,036) y preocupación por la situación financiera (p=0,048).

**Conclusión:**

se identificó una alta prevalencia de riesgo actual de suicidio entre los estudiantes de posgrado y una asociación significativa de este riesgo con variables demográficas, socioeconómicas, académicas y de salud.

## Introduction

In the last decade, researchers have turned their attention to the serious problems related to suicide among university students, especially undergraduate students^(^ 1 ^-^ 4 ^)^. However, more recently, evidence regarding the vulnerability to suicidal behavior among postgraduate students has drawn the attention of researchers, health professionals, professors and institutions responsible for this population in different countries such as the United States^(^ 5 ^)^, China^(^ 6 ^)^ and Brazil^(^ 7 ^)^, mainly due to the fact that *stricto sensu* postgraduate teaching has peculiarities and generates demands that exert great influence on the students’ lives, predisposing them to psychological distress and mental illness^(^ 7 ^-^ 8 ^)^.

In this context, suffering can have a negative impact on mental health, manifesting as malaise, feelings of anguish, stress, anxiety and tension, and may even take on more serious mental disorders, as well as suicide risk^(^ 7 ^-^ 8 ^)^.

The risk of suicide, regardless of the measure used, is determined based on elements that make up suicidal behavior, a *continuum* of events that permeates suicidal ideation, attempted suicide and the act itself^(^ 9 ^)^. A study developed in the United States of America verified the risk of suicide among postgraduate students and showed that 21.2% met the criteria for suicide risk^(^ 10 ^)^.

Regarding suicide, it is known that this is a multi-factorial phenomenon, related to the sociocultural context that can be determined by diverse factors such as demographic, socioeconomic and health factors, common both in the general population^(^ 9 ^)^ and in postgraduate students^(^ 5 ^-^ 8 ^)^. In particular, some academic factors related to postgraduate studies, such as long hours devoted to academic work, intense demands regarding the production of articles or of the thesis and/or dissertation, scarce resources for scientific funding, among others, have been described in the scientific literature as potentiators in the development of psychological distress^(^ 7 ^)^. However, despite these aspects, relatively little is known about the association of these factors related to college with the risk of suicide in this population^(^ 5 ^,^ 8 ^)^.

Given the context presented, this research starts from the hypothesis that there is an association between demographic, socioeconomic, health and academic factors and the risk of suicide among *stricto sensu* students in the Brazilian context. Furthermore, the fact is that the phenomenon is complex and multi-causal and the identification of associated factors in this population has the potential to contribute to filling the current scientific gap, as well as being able to subsidize interventions on university *campi* contributing preventively to the reduction in suicide risk and in the the harms caused by such a condition among Brazilian postgraduate students. Therefore, this study was developed with the objective of analyzing the factors associated with the risk of suicide in postgraduate students.

## Method

An analytical and cross-sectional study developed in a federal public university in the Brazilian Midwest region from August to November 2019.

The study was carried out with *stricto sensu* postgraduate students (master’s and doctorate) from all knowledge areas (agrarian sciences, human sciences, health sciences, applied social sciences, biological sciences, exact and earth sciences, multidisciplinary and linguistic, language and arts), enrolled on the four *campi* of the university, totaling 2,449 individuals at the time of the study. To determine the sample, a stratified probabilistic sampling method proportional to the size of the population was used, in which the strata were constituted by the four *campi*, considering a proportion of 50%, a sampling error of 4%, and a confidence level of 95%. Thus, a sample (n) of 482 participants was estimated. Predicting possible losses, this total was corrected for 565 postgraduate students, ensuring 85% coverage of the final sample^(^ 11 ^)^ with the following distribution: 480 students on the main campus, 35 on the second largest campus, 32 on the third, and 18 in the fourth. The study included all the individuals who were regularly enrolled during the data collection period, without applying exclusion criteria.

Four instruments were used for data collection: ^(^ 1 ^)^ To assess the current risk of suicide (dependent variable), Module C of the Mini International Neuropsychiatric Interview (MINI), version 5.0 was used, adapted for self-application. The instrument is translated into Brazilian Portuguese^(^ 12 ^)^ and validated for use in an adult population, with good psychometric performance^(^ 13 ^)^. It consists of five dichotomous questions (yes/no) that assess suicidal behavior in the last 30 days (four questions) and throughout life (one question). The score for risk stratification can vary from 0 to 33 points, with the possibility of being classified as low (1-5 points), moderate (6-9 points), and high (≥ 10 points) risk. For analysis, in this study, the following categorization was performed: no risk of suicide (0 points) and current risk of suicide (1-33 points); ^(^ 2 ^)^ The CAGE (Cut down, Annoyed by criticism, Guilty and Eye-opener) questionnaire was used to detect cases of alcohol dependence or abuse. It is an instrument composed of four dichotomous questions (yes/no), validated in Brazil, with good levels of sensitivity and specificity. Regarding the cutoff point, two or more affirmative answers indicate a situation of alcohol dependence/abuse^(^ 14 ^)^; ^(^ 3 ^)^ The Depression, Anxiety and Stress Scale (DASS-21) was used to investigate depressive, anxious and stress symptoms. It is an instrument composed of 21 questions, divided into three subscales (seven questions each), with answers on a four-point Likert scale. Each subscale provides an initial score that can vary from 0 to 21 points. Subsequently, this result was multiplied by two, according to the guidelines of the original authors, providing a general score for each subscale ranging from 0 to 42 points. According to this score, the perception of symptoms can be classified as normal, mild, moderate, severe and/or extremely severe. For the purposes of analysis in this study, the normal group was considered as “no symptoms” and the others as “presence of symptoms”. It is noteworthy that this instrument has been translated^(^ 15 ^)^ and validated for use in Brazilian university students^(^ 16 ^)^, with good internal consistency for each subscale^(^ 15 ^-^ 16 ^)^; ^(^ 4 ^)^ An instrument developed by the main researcher and validated in face and content by a group of six specialists in the areas of postgraduate studies and suicide, through the Content Validity Index (CVI). It consists of 42 questions containing demographic, socioeconomic, health and academic variables (which investigated interpersonal relationships and feelings about postgraduate studies, type of course and research development). The total CVI score of the instrument was calculated by dividing the total number of items considered relevant by the judges, by the total number of items on the instrument, which represented an agreement of 0.93.

The data were collected online, via email, using the Google® Forms tool. A list of all 2,449 *stricto sensu* postgraduate students was provided by the Pro-Rector of Postgraduate Studies at the university. Of this population, 565 subjects were randomly drawn, respecting proportionality by *campus*. All those selected in this stage received an invitation via email to participate in the study and a link to access the data collection instruments. Of these, the 26 students who refused to participate in the study or did not respond to the email sent up to three times, with an interval of one week, were replaced by the next subject on the list to reach the minimum sample. The Free and Informed Consent Form was made available online and, after reading it, the students answered if they agreed to participate in the study by clicking on the dialog box corresponding to “yes”, in addition to registering a valid email address in order to avoid duplication. It should be noted that there were no questionnaires with incomplete data (missing data).

The data were analyzed in a descriptive and inferential manner using the *Statistical Package for the Social Sciences* (SPSS) software, version 20. In the descriptive analysis, absolute, relative frequencies or prevalence values were presented. For the inferential analysis, the gross prevalence ratio (PR_g_) and Pearson’s chi-square test were used, with a significance level lower than 0.05 (p<0.05) and their respective 95% confidence intervals. For multiple analysis, variables with a p-value below 0.20 (p<0.20) were considered, and variables with a p-value below 0.05 (p<0.05) remained in the final model, with their adjusted prevalence ratios (PR_a_) and 95% confidence intervals.

The research complied with the norms of Resolution No. 466/2012 of the National Health Council, and is approved by the Health Research Ethics Committee of the Federal University of Mato Grosso under Opinion No.: 3,462,827 and CAAE: 13273119.3.0000.8124, July 2019.

## Results

A total of 565 postgraduate students participated in the study, with a prevalence of current suicide risk estimated at 40.18%. They had a median age of 30 years old, ranging from 20 to 59 years old, with a predominance of those who declared to have some faith practice (79.11%), to be concerned about the financial situation (72.92%), and to have a partner (51.68%). In the health variables, 54.34% presented symptoms of anxiety, 52.04% of depression, 50.27% of stress, and 35.58% reported using psychotropic drugs during postgraduate school, with or without medical prescription.

In relation to the academic variables: 65.84% of the postgraduate students were attending master’s degrees and 34.16% doctorate courses, 43.36% agreed that their research could be harmed by lack of funding, 20.00% agreed that the family relationship was hindered by the demands of postgraduation, and 13.98% did not have a good relationship with the technicians of the course, 11.86% with their colleagues, 10.44% with the advisor, and 10.09% with the professors of the postgraduate course; and 12.04% disagreed that their academic work was significant and inspiring.

Table 1 shows that the demographic and socioeconomic variables associated with the current risk of suicide were median age > 30 years old, not having any faith practice, being concerned about their financial situation (p<0.001, respectively), and marital status without a partner (p=0.005).

**Table 1:** Association between the current suicide risk and the demographic and socioeconomic variables of the *stricto sensu* postgraduate students from a federal university in the Midwest region. Cuiabá, Mato Grosso, Brazil, 2019

Variable	Current suicide risk			^†^95% CI	^‡^p-value
	Yes	No			
	n (%)	n (%)	*PR_g_		
Median age (Md=30)					
>30 years old	138 (47.92)	150 (52.08)	1.49	1.21; 1.84	<0.001
≤30 years old	89 (32.13)	188 (67.87)	1.00	-	-
Faith practice					
No	65 (55.08)	53 (44.92)	1.52	1.2; 1.86	<0.001
Yes	162 (36.24)	285 (63.76)	1.00	-	-
Concern about the financial situation					
Yes	188 (45.63)	224 (54.37)	1.79	1.34; 2.39	<0.001
No	39 (25.49)	114 (74.51)	1.00	-	-
Marital status					
No partner	126 (46.15)	147 (53.85)	1.33	1.09; 1.64	0.005
Has a partner	101 (34.59)	191 (65.41)	1.00	-	-

* PR_g_= Gross Prevalence Ratio;

† 95% CI = 95% Confidence Interval;

‡ p-value = Chi-square test

The associations between the current suicide risk and health variables were shown in Table 2, with statistical significance (p<0.001) for postgraduate students who used psychotropic drugs during the course or who were in current use, who used any illicit drugs throughout life, presented depression, anxiety and stress symptoms, and with p=0.004 for the alcohol abuse variable.

**Table 2:** Association between the current suicide risk and the health variables of the *stricto sensu* postgraduate students from a federal university in the Midwest region. Cuiabá, Mato Grosso, Brazil, 2019

Variable	Current suicide risk			^†^95% CI	^‡^p-value
	Yes	No			
	n (%)	n (%)	*PR_g_		
Use of psychotropic drugs during the course					
Yes	133 (66.17)	68 (33.83)	2.56	2.10; 3.13	<0.001
No	94 (25.82)	270 (74.18)	1.00	-	-
Current use of psychotropic drugs					
Yes	104 (71.23)	42 (28.77)	2.43	2.03; 2.91	<0.001
No	123 (29.36)	296 (70.64)	1.00	-	-
Use of illicit drugs					
Yes	80 (54.79)	66 (45.21)	1.56	1.28; 1.90	<0.001
No	147 (35.08)	272 (64.92)	1.00	-	-
Depression symptoms					
Present	182 (61.90)	112 (38.10)	3.73	2.81; 4.94	<0.001
Absent	45 (16.61)	226 (83.39)	1.00	-	-
Anxiety symptoms					
Present	180 (58.63)	127 (41.37)	3.22	2.44; 4.44	<0.001
Absent	47 (18.22)	211 (81.78)	1.00	-	-
Stress symptoms					
Present	164 (57.75)	120 (42.25)	2.58	2.03; 3.27	<0.001
Absent	63 (22.42)	218 (77.58)	1.00	-	-
Alcohol abuse					
Yes	33 (57.89)	24 (42.11)	1.52	1.18; 1.94	0.004
No	194 (38.19)	314 (61.81)	1.00	-	-
Search for mental health service while in postgraduate course					
No	61 (35.88)	109 (64.12)	0.85	0.68; 1.08	0.172
Yes	166 (42.03)	229 (57.97)	1.00	-	-

* PRg = Gross Prevalence Ratio;

† 95% CI = 95% Confidence Interval;

‡ p-value = Chi-square test

Table 3 shows a statistical association between the current risk of suicide and the academic variables that involved interpersonal relationships, such as having family relationships hindered by postgraduate demands, not having a good relationship with the colleagues in postgraduate studies (p<0.001, respectively), as well as with the advisor (p=0.004), the postgraduate professors (p=0.010) and the course technicians (p=0.041). Regarding the feelings about post-graduation, being unable to perform leisure activities due to the demands of postgraduation (p=0.014), suffering discrimination in the postgraduate course (p=0.004), not considering the academic work meaningful and inspiring, not being optimistic about future professional perspectives (p<0.001, respectively), and not believing that they would complete postgraduate school in the regular time (p=0.001) were associated with suicide risk.

**Table 3:** Association between the current suicide risk and the academic variables (interpersonal relationship and feelings about postgraduate studies) of the *stricto sensu* postgraduate students from a federal university in the Midwest region. Cuiabá, Mato Grosso, Brazil, 2019

Variable	Current suicide risk			^†^95% CI	^‡^p-value
	Yes	No			
	n (%)	n (%)	*PR_g_		
Family relationship hindered by demands of postgraduate studies					
Agree	65 (57.52)	48 (42.48)	1.60	1.31; 1.96	<0.001
Disagree	162 (35.84)	290 (64.16)	1.00	-	-
Good relationship with postgraduate colleagues					
Disagree	42 (62.69)	25 (37.31)	1.69	1.36; 2.10	<0.001
Agree	185 (37.15)	313 (62.85)	1.00	-	-
Good relationship with advisor					
Disagree	34(57.63)	25 (42.37)	1.51	1.18; 1.93	0.004
Agree	193 (38.14)	313 (61.86)	1.00	-	-
Good relationship with postgraduate professors					
Disagree	32 (56.14)	25 (43.86)	1.46	1.13; 1.89	0.010
Agree	195 (38.39)	313 (61.61)	1.00	-	-
Good relationship with course administrative technicians					
Disagree	40 (50.63)	39 (49.37)	1.32	1.03; 1.68	0.041
Agree	187 (38.48)	299 (61.52)	1.00	-	-
Being unable to perform leisure activities due to the demands of postgraduate studies					
Agree	69 (48.94)	72 (51.06)	1.31	1.07; 1.62	0.014
Disagree	158 (37.26)	266 (62.74)	1.00	-	-
Discrimination suffered in the postgraduate course					
Agree	33 (57.89)	24 (42.11)	1.52	1.18; 1.94	0.004
Disagree	194 (38.19)	314 (61.81)	1.00	-	-
Meaningful and inspiring academic work					
Disagree	42 (61.76)	26 (38.24)	1.66	1.33; 2.07	<0.001
Agree	185 (37.22)	312 (62.78)	1.00	-	-
Optimism about future professional perspectives					
Disagree	131(51.17)	125(48.83)	1.65	1.34; 2.02	<0.001
Agree	96 (31.07)	213 (68.93)	1.00	-	-
Course completion in regimental time					
Disagree	63 (53.85)	54 (46.15)	1.47	1.19; 1.81	0.001
Agree	164 (36.61)	284 (63.39)	1.00	-	-

* PR_g_= Gross Prevalence Ratio;

† 95% CI = 95% Confidence Interval;

‡ p-value = Chi-square test

Table 4 shows a statistical association between the current risk of suicide and the academic variables that involved the course/program and the development of research such as being enrolled in the doctoral course (p=0.002), away from postgraduate school for physical health problems, psychological distress or due to statutory leave (maternity) (p=0.006), being under pressure to produce material for publication, having difficulty writing their theses/dissertations, not having productive meetings with the advisor, research development hindered by lack of funding (p<0.001, respectively) and not having any subsidy from the disciplines offered by the course for the development of research (p=0.018).

**Table 4:** Association between the current suicide risk and the academic variables (course and research development) of the *stricto sensu* postgraduate students from a federal university in the Midwest region. Cuiabá, Mato Grosso, Brazil, 2019

Variable	Current suicide risk			^†^95% CI	^‡^p-value
	Yes	No			
	n (%)	n (%)	*PR_g_		
Enrolled *stricto sensu* course					
PhD	60 (31.09)	133 (68.91)	0.69	0.55; 0.88	0.002
Master's degree	167 (44.89)	205 (55.11)	1.00	-	-
Enrollment situation					
Away due to problems	10 (76.92)	3 (23.08)	1.96	1.43; 2.68	0.006
Active	217 (39.31)	335 (60.69)	1.00	-	-
Pressure to produce material for publication					
Agree	140 (49.12)	145 (50.88)	1.58	1.28; 1.95	<0.001
Disagree	87 (31.07)	193 (68.93)	1.00	-	-
Difficulty writing thesis/dissertation					
Agree	109 (53.43)	95 (46.57)	1.63	1.34; 1.99	<0.001
Disagree	118 (32.69)	243 (67.31)	1.00	-	-
Productive meetings for research with the advisor					
Disagree	57 (58.16)	41 (41.84)	1.60	1.30; 1.96	<0.001
Agree	170 (36.40)	297 (63.60)	1.00	-	-
Research development hindered by lack of funding					
Agree	122 (49.80)	123 (50.20)	1.52	1.24; 1.85	<0.001
Disagree	105 (32.81)	215 (67.19)	1.00	-	-
Subsidy of disciplines in the development of research					
Disagree	86 (47.25)	96 (52.75)	1.28	1.05; 1.57	0.018
Agree	141 (36.81)	242 (63.19)	1.00	-	-
Activity parallel to postgraduate studies					
No	117 (43.33)	153 (56.67)	1.16	0.95; 1.42	0.143
Yes	110 (37.29)	185 (62.71)	1.00	-	-
Scholarship					
Yes	117 (43.98)	149 (56.02)	1.20	0.98; 1.46	0.082
No	110 (36.79)	189 (63.21)	1.00	-	-

* PR_g_= Gross Prevalence Ratio;

† 95% CI = 95% Confidence Interval;

‡ p-value = Chi-square test

Table 5 shows, after multiple regression, the variables that remained in the final model, with median age > 30 years old (p=0.029), absence of faith practice (p=0.015), depression symptoms (p<0.001), anxiety symptoms (p=0.018), use of psychotropic drugs during the course (p<0.001), not having a meaningful and inspiring academic work (p=0.013), not having a good relationship with the postgraduate colleagues (p=0.033), having the family relationship hindered by the demands of postgraduate studies (p=0.036), and concern about the financial situation (p=0.048). The enrolled *stricto sensu* course variable remained in the final model only as an adjustment variable for this model, which improves the explanatory power, although it was not statistically significant.

**Table 5:** Variables of the final model and prevalence ratio adjusted by Robust Poisson regression (RP_a_) associated with the current risk of suicide, with their respective 95% confidence intervals (CIs) and p-values. Cuiabá, Mato Grosso, Brazil, 2019.

Variable	Category	*PR_a_	^†^95% CI	^‡^p-value
**Median age (Md=30)**	**>30 years old**	**1.22**	**1.02 to 1.47**	**0.029‡**
	≤30 years old	1.00	-	-
Faith practice	No	1.25	1.04 to 1.49	0.015‡
	Yes	1.00	-	-
Depression symptoms	Present	2.04	1.46 to 2.85	<0.001‡
	Absent	1.00	-	-
Anxiety symptoms	Present	1.46	1.07 to 2.00	0.018‡
	Absent	1.00	-	-
Use of psychotropic drugs during the course	Yes	1.77	1.44 to 2.17	<0.001‡
	No	1.00	-	-
Meaningful and inspiring academic work	Disagree	1.33	1.06 to 1.67	0.013‡
	Agree	1.00	-	-
Good relationship with postgraduate colleagues	Disagree	1.25	1.02 to 1.54	0.033‡
	Agree	1.00	-	-
Family relationship hindered by the demands of postgraduate studies	Agree	1.19	1.01 to 1.40	0.036‡
	Disagree	1.00	-	-
Concern about the financial situation	Yes	1.30	1.01 to 1.69	0.048‡
	No	1.00	-	-
Enrolled *stricto sensu* course	PhD	0.83	0.68 to 1.03	0.084
	Master's degree	1.00	-	-

* PR_a_= Prevalence Ratio adjusted by the Robust Poisson regression model with selection of variables by the backward method;

† CI = Confidence Interval;

‡ Significant at the 5% level. Model p-value<0.05

## Discussion

The current risk of suicide (last 30 days) for the sample in this study was 40.18% and can be considered a high indicator when compared to the study developed with American postgraduate students, which obtained a risk prevalence of 21.2%^(^ 10 ^)^. However, the differences evidenced between these percentages must be analyzed considering that the research studies used different risk assessment instruments and were carried out in populations and regions with different sociocultural characteristics.

Regarding the associated factors, two sociodemographic variables are related in a statistically significant manner to the current risk of suicide: median age and faith/religiousness practice.

For being a universal phenomenon, suicide affects individuals of all ages and, despite epidemiological evidence showing that the highest mortality rates due to suicide are concentrated in two specific age groups, older adults and young adults (15 – 29 years old)^(^ 17 ^)^, suicidal behaviors among adults and middle-aged individuals (30 – 50 years old) are not uncommon. In this research, students over the age of 30 were at greater current risk for suicide than their younger peers. It is noteworthy that, although there are no clear explanations in the literature to justify these differences, the authors believe that postgraduate older-aged students may have some characteristics such as suffering greater social and family pressure to enter the labor market or experience greater conflicts to conciliate academic, family and social activities that can compromise their mental health and expose them to an increased risk for suicidal behavior. However, in order to confirm this hypothesis, new studies need to be developed in this population group.

In relation to faith practices, these have been highlighted as important protective factors against suicide, acting as a coping mechanism and positively influencing the way people face stressors, distress and personal crisis situations. Spirituality can provide an increased sense of purpose and meaning in life, elements associated with greater resilience, self-confidence and resistance to stress related to diseases^(^ 18 ^)^. Such considerations may justify the association obtained between the lack of faith/religiousness practice and the higher current risk of suicide among the postgraduate students in this study.

In addition to the sociodemographic variables, the presence of depressive and anxiety symptoms was also associated with the current risk of suicide among postgraduate students.

In this regard, it is known that the *stricto sensu* postgraduate program is a period marked by intense requirements and pressures in a competitive environment with demands for production and publication, demanding long hours in research, reading scientific literature, writing reports and scientific communications forcing the students to renounce their social life and leisure time. This large volume of work associated with deadlines, generally short, for the delivery of productions, the intense routine of studying and preparing materials, limited resources, little institutional support for carrying out research and fragile and conflicting social relationships (between peers and advisors) are elements that contribute to the constitution of a pathological environment with the potential of developing symptoms of mental illness such as depression and anxiety^(^ 19 ^)^.

The association between the clinical condition of depressive symptoms and suicidal behavior has been widely described in the scientific literature, in several population segments^(^ 20 ^-^ 22 ^)^, with an evident positive association between the two phenomena that often coexist and influence each other^(^ 23 ^)^. A number of studies carried out with postgraduate students have shown that this is a population at risk for depressive symptoms, frequently presenting prevalence indicators considerably higher than those of the general population^(^ 24 ^)^.

Regarding anxiety disorders, in isolation, they are not always associated with suicide but, when they occur in conjunction with depressive symptoms, the risk for suicide and/or attempted suicide tends to increase and be significant, showing that depression can act as a mediator between anxiety and suicidal behavior^(^ 23 ^,^ 25 ^)^. A study with 2,279 postgraduate students, 90% PhD and 10% master’s, interviewed at 234 institutions in 26 countries from different areas, showed proportions of 39% depression and 41% moderate to severe anxiety among the students, demonstrating these conditions in the lives of most postgraduate students^(^ 24 ^)^.

It was also evidenced that students who used psychotropic drugs at some point during the course, with or without a medical prescription, were at a higher risk of suicide. It is assumed that the use of psychotropic drugs during the postgraduate course has increased due to the increase in psychological problems in this population, such as anxiety, depression, insomnia and nervous crisis. Thus, in an attempt to minimize the problem, many postgraduate students resort to the use of these drugs without a medical prescription^(^ 7 ^)^.

In addition, there is the possibility of consuming certain psychoactive substances, mainly those manufactured and sold legally, with the aim of improving academic performance^(^ 26 ^)^. Although no studies were found discussing this fact in postgraduate students, it is considered that they may use these drugs because they are inserted in an academic environment in which good performance and high productivity are required.

Regarding the academic factors evaluated, it was verified that those who did not have a significant and inspiring academic work had a higher current risk of suicide. Academic work and the organizational context are significant predictors of the mental health of PhD students^(^ 27 ^)^. When the postgraduate student spends a lot of time and energy on a research that has no apparent usefulness, this fact can trigger a cycle of self-perpetuation of discouragement and disengagement, making the researcher more susceptible to mental illness^(^ 28 ^)^.

As for the relationship of postgraduate students with their colleagues, those who did not have a good relationship obtained a greater association with the current risk of suicide. Adult life can be characterized by a time when friendships are neglected as a result of work and family life, among other demands, which can repress their potential as support against stress^(^ 29 ^)^. Having a good relationship with peers becomes a protective factor against suicide risk in postgraduate students as affective interpersonal relationships help in coping with stressful life events^(^ 9 ^,^ 30 ^)^. Interpersonal interactions with academic peers tend to be those that occur most frequently in the university context, since postgraduate students usually interacts more with their colleagues than with the professors or the advisor^(^ 31 ^-^ 32 ^)^. In addition to frequency, relationships with peers differ from those established with other academic subjects due to their directionality, conditioned by power and knowledge relations implicit in the academic environment. While, in general, the interactions with the advisor and the professor are vertical, denoting the position of superiority in which they are in the academic hierarchy, the relationship`s with peers remain horizontal, marked by sharing knowledge and experiences and greater potential in promoting health and well-being^(^ 31 ^-^ 32 ^)^.

With regard to the findings in relation to the impaired family relationship facing the demands of postgraduate school, the work-family conflict is an important predictor of psychological distress and exposes the increased risk of common psychiatric disorder in doctoral students (p<0.001)^(^ 27 ^)^. The impairment in the relationship with the family of those who carry out academic work can be due to a sum of factors which are characteristic of the academic routine^(^ 33 ^)^, characterized in postgraduate school by devoting up to 80 weekly hours of activity, including weekends and holidays^(^ 8 ^)^. In this sense, psychological distress becomes present to the extent that there is an imbalance between the time devoted to the professional/academic and personal/family life^(^ 33 ^)^.

The association between the current risk of suicide and financial concerns, evidenced in the present study, is not a recent finding in the study of suicide. Some research studies have corroborated this association over the past decades, showing monetary losses and financial stress as one of the main factors for suicidal behaviors^(^ 34 ^-^ 35 ^)^. According to these studies, in general, suicide risk in these situations can arise impulsively in a moment of abrupt and unexpected crisis (loss of a job, loss of financial reserves) or from the individual’s difficulties to healthily deal with these stressors^(^ 34 ^-^ 35 ^)^. The relation between financial stress and impaired mental health/increased risk of suicide in the general population^(^ 35 ^)^ has been extensively explored in the scientific literature; however, little is known about this relation in some specific contexts, such as between undergraduate and postgraduate students.

Among national and international studies that measured factors associated with the stress experienced by postgraduate students, concerns have been raised about the lack of financial resources as one of the main sources of stress in this population^(^ 8 ^,^ 19 ^,^ 36). Several courses require students to devote themselves exclusively to carrying out their full-time research activities, forcing many to leave their jobs, resign or remain unemployed to enter the academic segment, which can represent a reduction in family income^(^ 36 ^)^.

It is noteworthy that receiving scholarships is not a guarantee of financial security for postgraduate students. The scholarship is understood as a salary with values stagnated for five years, does not sufficiently serve all those who request it and, even when obtained, the amount received is low and is usually not enough to cover all the needs. In Brazil, the last scholarship readjustment took place in 2013, with values not in line with the high degree of specialization and dedication required for stricto sensu postgraduate activities and, usually, below what could be earned outside the university^(^ 37 ^)^. Currently, based on 2020, the scholarship offered by the Coordination for the Improvement of Higher Level Personnel (*Coordenação de Aperfeiçoamento de Pessoal de Nível Superior*, CAPES) is equivalent to 1.4 and 2.1 minimum wages for master’s and PhD degrees, respectively. The aforementioned aid does not offer a 13th salary, vacation or contributes to retirement purposes, and the devaluation of the purchase power of the scholarship is evident when compared to the same value a decade ago, which was equivalent to 2.9 and 3.4 minimum wages for master›s and doctorate degrees, respectively^(^ 7 ^)^.

Postgraduate students in a situation of financial insecurity are very likely to evade their course and, when this does not happen, they are more susceptible to mental distress, increased prevalence of anxiety and depression symptoms, and perception of feelings of inadequacy, hopelessness and helplessness. These feelings have the potential to trigger suicidal (risk) behaviors of different orders and may be triggers for suicidal ideation or suicide attempts^(^ 36 ^)^.

In relation to the limitations of the study, the difficulty in comparing the results with other realities in different contexts in the population of postgraduate students is cited, since the few data described in the scientific literature are related to aspects of suicidal behavior, such as presence of ideation and attempted suicide^(^ 5 ^,^ 19 ^)^ and not to the risk itself. In addition, it is pointed out that the research subjects are representative of a single Brazilian university, making it difficult to generalize the results to other contexts and/or regions of the country.

Finally, there are few national and international studies on suicidal behavior and/or suicide risk among master’s and PhD students^(^ 8 ^)^ and several gaps, mainly on associated factors, need to be adequately answered. In view of this scenario, the results of this study contribute to broaden the understanding of how certain aspects (demographic, socioeconomic, health and academic) are associated with the suicide risk in a sample of Brazilian students, and they present evidence that indicates the vulnerability of this population for suicidal behavior. They also allow for the constitution of an important situational diagnosis for postgraduate institutions and programs, especially the national ones, to implement strategies, such as lectures and institutional training programs to identify suicide risk and understand suicidal behavior, successful experiences carried out in other university contexts^(^ 38 ^-^ 40 ^)^, although based on specific risk factors for this population.

## Conclusion

In the present study, a high prevalence of current suicide risk was identified among stricto sensu postgraduate students and that variables such as age > 30 years old, lack of faith practice, depression and anxiety symptoms, use of psychotropic drugs during the course; not having a meaningful and inspiring academic work, not having a good relationship with the colleagues at the postgraduate level, having a family relationship impaired by the demands of the postgraduate program, and concern about the financial situation were associated in a statistically significant manner with this risk. Up to the present day, the findings of this study are unprecedented, which reinforces the need for further research studies with this population, even with different methodological designs, helping to identify and better understand suicidal behavior among postgraduate students.
